# New Horizons in Higher Education: Examining the Mental Well-Being of Medical & Health Sciences Students Through the Use of Artificial Intelligence Based Chatbot Platforms in the United Arab Emirates – A Cross-Sectional Comparative Study

**DOI:** 10.12688/f1000research.166372.2

**Published:** 2025-10-06

**Authors:** Eman Abdelaziz Rashad Dabou, Fatma Magdi Ibrahim, Mustafa Faisal Haimour, Aya Saleh, Richard Mottershead

**Affiliations:** 1 RAK College of Nursing,, Ras Al Khaimah Medical and Health Sciences University, Ras Al Khaimah, United Arab Emirates; 2 Faculty of Nursing, Alexandria University, Alexandria, Egypt; 3 Faculty of Nursing, Mansoura University, Mansoura, Dakahlia Governorate, Egypt; 4 Faculty of Nursing, College of Health Sciences, University of Sharjah, Sharjah, United Arab Emirates; 5 College of Nursing, University of Baghdad, Baghdad, Iraq; 6 Sakina, Behavioural Science Institute, SEHA, SEHA, Al Ain, United Arab Emirates

**Keywords:** Artificial Intelligence, Chatbot, Students, Mental Health & Well-Being

## Abstract

**Background:**

Barriers to mental-health care include limited resources and workforce, access constraints, and stigma. Artificial-intelligence (AI)–enabled chatbots may offer low-threshold support.

**Methods:**

Cross-sectional correlational (comparative) study in one private health-sciences university in the UAE. Proportional stratified random sampling across four colleges yielded *n* = 298 undergraduates. Instruments: (i) Socio-Demographic Questionnaire; (ii) researcher-developed AI Chatbot Usability questionnaire (content validated by a bilingual specialist); and (iii) Depression Anxiety Stress Scale-21 (DASS-21; established reliability/validity). Questionnaires were administered face-to-face with standardized instructions.

**Results:**

206/298 (69.1%) had ever used an AI chatbot; most used Snapchat AI (76.9%), followed by ChatGPT/Bard (23.4% each). Overall, 57.0% had moderate-to-extremely-severe depression, 68.5% anxiety, and 33.6% stress. Users had higher odds of moderate-to-extremely-severe anxiety and depression than non-users. In multivariable models, higher depression (OR = 1.022; 95% CI 1.01–1.085; *p*<0.001) and anxiety (OR = 1.05; 95% CI 1.01–1.21; *p*<0.001) independently predicted chatbot use; stress did not.

**Conclusion:**

Among UAE health-sciences students, AI-chatbot use is common and associated with higher depression/anxiety severity; this likely reflects help-seeking rather than causation. Universities should integrate early, stigma-sensitive supports, potentially including regulated, evidence-based chatbot tools—within stepped-care services.

## Introduction

The global mental health care system is currently facing significant challenges and a need to explore new treatment approaches inclusive of digital healthcare strategies. The World Health Organization states that one in four individuals may experience mental illness at some stage in their lives ( Pontes et al., 2021). With growing concerns of an increase in the vulnerability of adolescent mental health post-COVID 19 pandemic by Wright et al. (2024) there is a pressing need to identify new modes of treatment accessible and familiar with this generation. Indeed, the World Health Organisation ( WHO, 2017) explains that an overreliance of hospital-based care has the potential to create barriers which consequently may impact the identification and recovery from mental illness. The authors seek to expand the knowledge base around AI chatbot use within the Middle East due to a growing need for comparative analyse with global studies. The authors highlight the research of Dattani (2021) that reports that mental health conditions in the Middle East have remained relatively consistent over the past two decades. Albeit, that mental health conditions are increasing as a share of the total disease burden. Worryingly, research by Al Habeeb et al. (2023) states that the Middle East and North Africa (MENA) region forms the global concentration for the proportion of mental health disorders as a disproportional share of the total disease burden. In support Dattani (2021) explains that in Kuwait, Jordan, Oman, and Qatar the percentage of reported mental health conditions as a share of the total disease burden is more than double the global average of 5%. The study’s authors articulate that the post-COVID-19 era has necessitated a need for healthcare leaders to continue to examine new innovative strategies inclusive of AI for a population with a lived experience of a global humanitarian crisis. Al Habeeb et al. (2023) supports this opinion in that adolescents are at risk of mental illness and with a higher burden of noncommunicable diseases. Indeed, mental disorders remain the primary source of health-related economic distress globally ( Ransome et al., 2022). Depression and anxiety are the predominant causes, impacting around 322 million (depression) and 264 million (anxiety) individuals worldwide ( Levant et al., 2022).

While global estimates highlight substantial depression and anxiety burden among students, robust Middle East/Gulf data particularly on AI-chatbot use for mental well-being, remain scarce. This study addresses that gap by describing usage patterns and their associations with DASS-21 severity in a UAE health-sciences cohort, adding region-specific evidence to predominantly Western literature.

In the UAE/MENA context, access barriers include constrained specialist capacity, uneven service availability, and stigma around help-seeking. Low-threshold digital tools, especially chatbots embedded in widely used platforms, may reduce friction, enable privacy, and increase timeliness of support for students.

The primary barriers to effective and comprehensive treatment are insufficient resources and competent medical personnel, alongside social discrimination, stigma, and marginalization. However, there is a beacon of hope. Information technology tools, particularly AI-enabled technologies, are emerging as a promising solution for longstanding difficulties such as societal stigma. These technologies are expected to provide more accessible, cost-effective, and potentially fewer stigmatizing alternatives to traditional mental health treatment models ( Williamson et al., 2022). It is theorized that by reducing the stigma associated with mental health, AI has potential for paving the way for a more supportive and encouraging environment for those in need and those whose preference maybe digital health themed. The author’s aim was to conduct research that expands the knowledge of AI chatbot use to support mental well-being within the Middle East and specifically in the United Arab Emirates.

## Background

Artificial intelligence (AI) has had a significant impact on our daily lives. Gupta et al. (2023) explains that the causality of these enhancements is due to the advancement of artificial intelligence in recent years. Conversational agents, or chatbots, are software systems featuring a conversational user interface. They can be classified as open-domain if they engage with users on any topic or task-specific if they assist with a particular activity. The subsequent ideas are fundamental to chatbot technology. Chatbots are AI-driven software systems capable of engaging in natural language communication with individuals through text or voice interactions ( Lee et al., 2024; Paay et al., 2022). This technology has continuously evolved and is presently employed in digital assistants like Apple’s Siri, Yandex’s Alice, Amazon’s Alexa, and other virtual assistants, in addition to consumer interfaces in electronic commerce and online banking ( Nirala et al., 2022).

Depression, anxiety, and stress are prevalent among university students and impact the lives of many within their academic journey, and can lead to poor academic performance, unhealthy interpersonal relationships ( Lee et al., 2020), and sadly, a low quality of life ( Zhong et al., 2019). Mobile-based therapy chatbots are increasingly being used to help young adults who suffer from depression ( Guo et al., 2020; Sheldon et al., 2021). As more and more people are interacting with computers, Chabot is becoming increasingly popular. Major tech firms including Microsoft, Google, Amazon, and Apple, have all released “personal digital assistants” or “smart speakers” that serve as platforms for chatbots (also known as voicebots) in 2016, which has been dubbed “The rise of the Chabot”. When compared to more traditional means of human-computer connection, chatting with a Chabot is likely to feel more natural and intuitive because it mimics human contact.

As Artificial intelligence (AI) technology has advanced rapidly over the past decade, more and more publications have begun to acknowledge AI’s importance in Internet-based Psychological Interventions. Gratzer and Goldbloom (2020) and Vaidyam et al. (2019) found that AI chatbots can more closely mimic human therapists. Even though most universities offer free therapy for students, many students refuse to seek help when they are suffering from mental health issues due to the reason of low perceived need ( Andrade et al., 2014), attitude barriers ( Andrade et al., 2014; Neathery et al., 2020), and the lack of mental health education ( Neathery et al., 2020). Chabot could be a scalable solution that provides an interactive means of engaging users in behavioral health interventions driven by artificial intelligence. Although some Chabot platforms have shown promising early efficacy results, there is limited information about how people utilize these systems. Understanding the usage patterns of a Chabot for depression, anxiety, and stress among medical and health sciences students represents a crucial step towards improving Chabot’s design and providing information about Chabot’s strengths and limitations. Therefore, this study aimed to identify the relationship between the utilization of the Artificial Intelligence Chabot and Stress, Anxiety, and Depression levels among Medical and Health Sciences University Students within the United Arab Emirates.

### Research questions

RQ1. What are the frequencies of using the Chabot among medical and health sciences university students?

RQ2. What are the reasons for the usage of AI Chabot to cope with depression, anxiety, and stress among Medical and Health Sciences Students?

RQ3. Is there a relation between the usage of AI Chabot and depression, anxiety, and stress among Medical and Health Sciences University Students?

RQ4. Is there a difference between the group who is using Chabot and the one who does not about depression, anxiety, and stress levels?

## Methods

### Design

A quantitative, descriptive comparative research design was used in this study.

### Setting and participants

The study was undertaken at a private health-sciences university in the UAE comprising four colleges (Medicine, Dentistry, Pharmacy, Nursing). The sampling frame included all enrolled undergraduates in these colleges (population totals available by college; total *N* = 1,309: Medicine = 530; Dentistry = 298; Pharmacy = 123; Nursing = 358). We applied proportional stratified random sampling to ensure representation from each college using the allocation: nh=n×Nh/n. The sample achieved ( *n* = 298) matched the planned allocations (Medicine = 120; Dentistry = 68; Pharmacy = 28; Nursing = 82). Inclusion criterion was consented to participate.

### Sample size

Assuming a conservative proportion (p = 0.50), 95% confidence (z = 1.96), and 5% absolute precision (e = 0.05) with finite-population correction for *N* = 1,309, the required sample size is: 297

$$n=\frac{Nz^{2}p(1-p)}{e^{2}(N-1)+z^{2}p(1-p)}$$

which aligns with the achieved n = 298

### Data collection

A face-to-face survey was carried out to collect the data. The participants took approximately 10-15 minutes to complete the questionnaire, and the duration of data collection was two months. To collect the data three tools were used. The correspondence/final author is a licensed mental health practitioner within the United Arab Emirates and was able to ensure rigor within the data collection process.

### Instruments

***Tool I*** **: Socio-Demographic Characteristics Questionnaire:** This questionnaire includes questions on college, gender, age, nationality, and year of the study.

***Tool II*: AI Chatbot Usability questionnaire:** researcher-developed measure of exposure, frequency, platform(s), and motivations. Content validity was established by a bilingual specialist. *Operational definition added:* “frequent use” was defined a priori as ≥4 days/week **or** ≥5 interactions/week in the past month; primary analyses used “ever vs never,” with “frequent use” flagged for sensitivity analyses. Motivations included convenience/24-7 access, privacy/stigma avoidance, familiarity, and perceived social support.

***Tool III*** **: Depression Anxiety Stress Scale 21 (DASS-21).** The DASS-21 ( Lovibond and Lovibond, 1995) is standardized measure of depression, anxiety, and stress (7 items/scale) with established validity and reliability reported from Hispanic American, British, and Australian adults. Lovibond and Lovibond (1995) designed this tool to measure the emotional states of depression, anxiety, and stress through this set of three self-report scales. Seven items are sub-divided into three scales that collectively allow the DASS-21 tool to assess mental well-being. The first scale focuses on depression and is used to assess inertia, hopelessness, devaluation of life dysphoria, self-deprecation, lack of interest/involvement, and anhedonia. The second scale focuses on anxiety and assesses anxious effect, subjective experience, situational experience, muscle effects, and autonomic arousal. It should be noted that there are reports of the stress scale being sensitive to levels of chronic non-specific arousal ( Lovibond and Lovibond, 1995). This third scale assesses the participants ability to relax, recorded impatience, level of agitation, irritability and signs of over-reactivity. The final stage of the process is a holistic assessment, created through review of the calculated scores for depression (scale one), anxiety (scale two), and stress (scale three) are calculated through the accumulative score before progressing on to data analysis.

### Data analysis and management

Analyses used SPSS v28. DASS-21 subscales were categorized using standard cutoffs to “normal-to-mild” vs “moderate-to-extremely severe.” Associations between DASS categories and demographics/AI-use were examined with chi-square tests. Logistic regression modelled odds of AI-chatbot use adjusting for demographics and DASS severities; results are reported as ORs with 95% CIs. Internal consistency was assessed by Cronbach’s α for DASS-21 subscales and Tool II. Exploratory note: future collections will capture interaction mode (text/audio) to permit moderation analyses by severity. Two-tailed p<0.05 signified statistical significance.

## Results

### Demographic characteristics

Table 1 presents the demographic characteristics of the study participants. Most participants were female (N = 236, 79.2%), with a mean age of 20.9 ± 2.5 years. The most significant proportion of participants was from the College of Medicine (N = 120, 40.3%), followed by the College of Nursing (N = 82, 27.5%), Dental (N = 68, 22.8%), and Pharmacy (N = 28, 9.4%). Regarding the year of study, the highest percentage was in the first year (N = 107, 35.9%), followed by the third (N = 84, 28.2%), fourth (N = 73, 24.5%), fifth (N = 15, 5.0%), and second (N = 19, 6.4%) years ( Table 1).

**Table 1.** Demographic characteristics.

Demographic variables		N	%
**Gender**	**Female**	236	79.2%
	**Male**	62	20.8%
**Age**	**Mean ± SD**	20.9 ± 2.5	
	**Median, (IQR)**	21.8 (3)	
**Collage**	**Dental**	68	22.8%
	**Medicine**	120	40.3%
	**Nursing**	82	27.5%
	**Pharmacy**	28	9.4%
**Year of Study**	**Fifth**	15	5.0%
	**First**	107	35.9%
	**Fourth**	73	24.5%
	**Second**	19	6.4%
	**Third**	84	28.2%

This table provides the demographic breakdown of students by gender, age, college, and year of study. Data are presented as n number and % = percentage.

### AI chatbot usage

**Figure 1.**
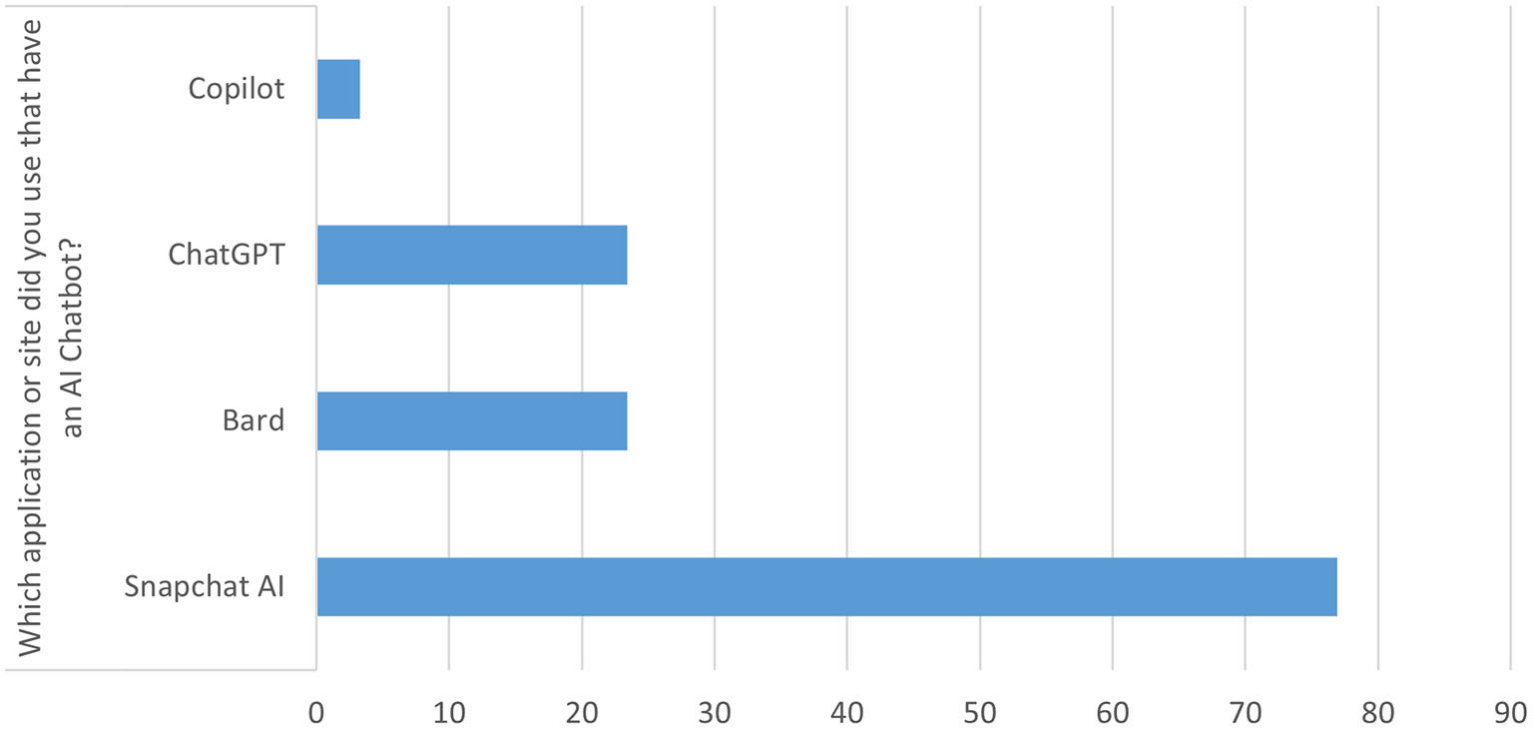
AI applications used.

Table 2 presents the usage of AI chatbots among the students. A total of 206 participants (69.1%) reported having ever spoken with an artificially intelligent chatbot. The most used AI chatbot applications were Snapchat (N = 230, 76.9%), followed by ChatGPT and Bard (N = 70, 23.4% each), and Copilot (N = 10, 3.3%): Table 2, Figure 1.

**Table 2.** AI usage among the students academic years (p < 0.001 for all).

Chatbot usage		N	%
Have you ever spoken with an artificially intelligent chatbot?	Yes	206	69.1%
	No	92	30.9%
Which application or site did you use that has an AI Chatbot?	Snapchat AI	230	76.9
	ChatGPT	70	23.4
	Copilot	10	3.3
	Bard	70	23.4

This table displays data on students' interactions with AI chatbots, including usage rates and platforms used. It also provides the demographic breakdown of students by gender, age, college, and year of study. Data are presented as n = number and % = percentage.

### Reasons for usage the AI chatbots

**Figure 2.**
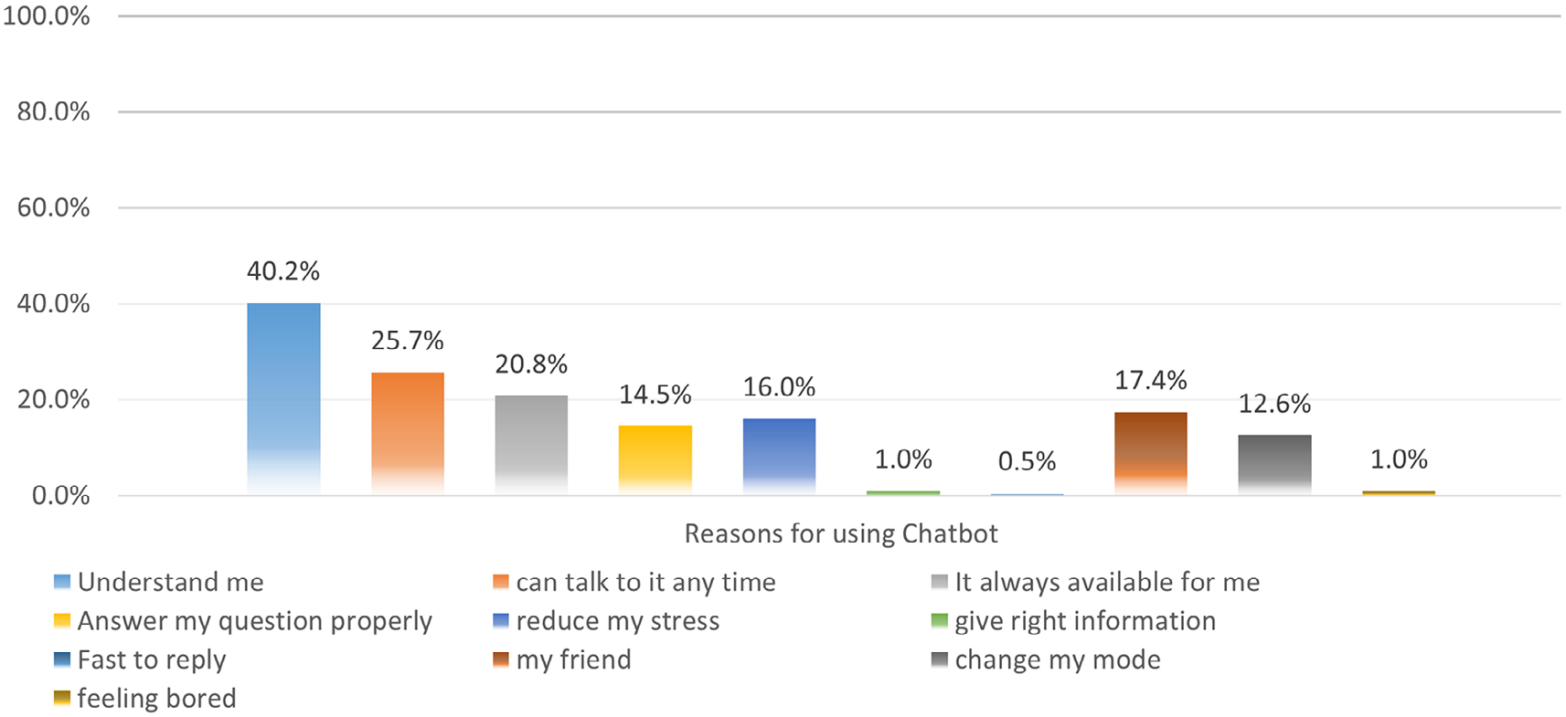
Reasons for usage the AI chatbots.

Nearly half of the study’s participants (40%) mentioned using AI chatbots because they are familiar with the interface with the platform and feel have a familiarity and understanding of the systems, while 25.7% reported that they can access them anytime. 20.8% found that the AI chatbot is always available to them. 17.4% felt a relationship akin to the platform representing a friend, and 16% found that it has a positive impact on reducing their stress levels. Figure 2 outlines the engagement with the A. I platforms.

### Depression, anxiety, and stress among participants

**Figure 3.**
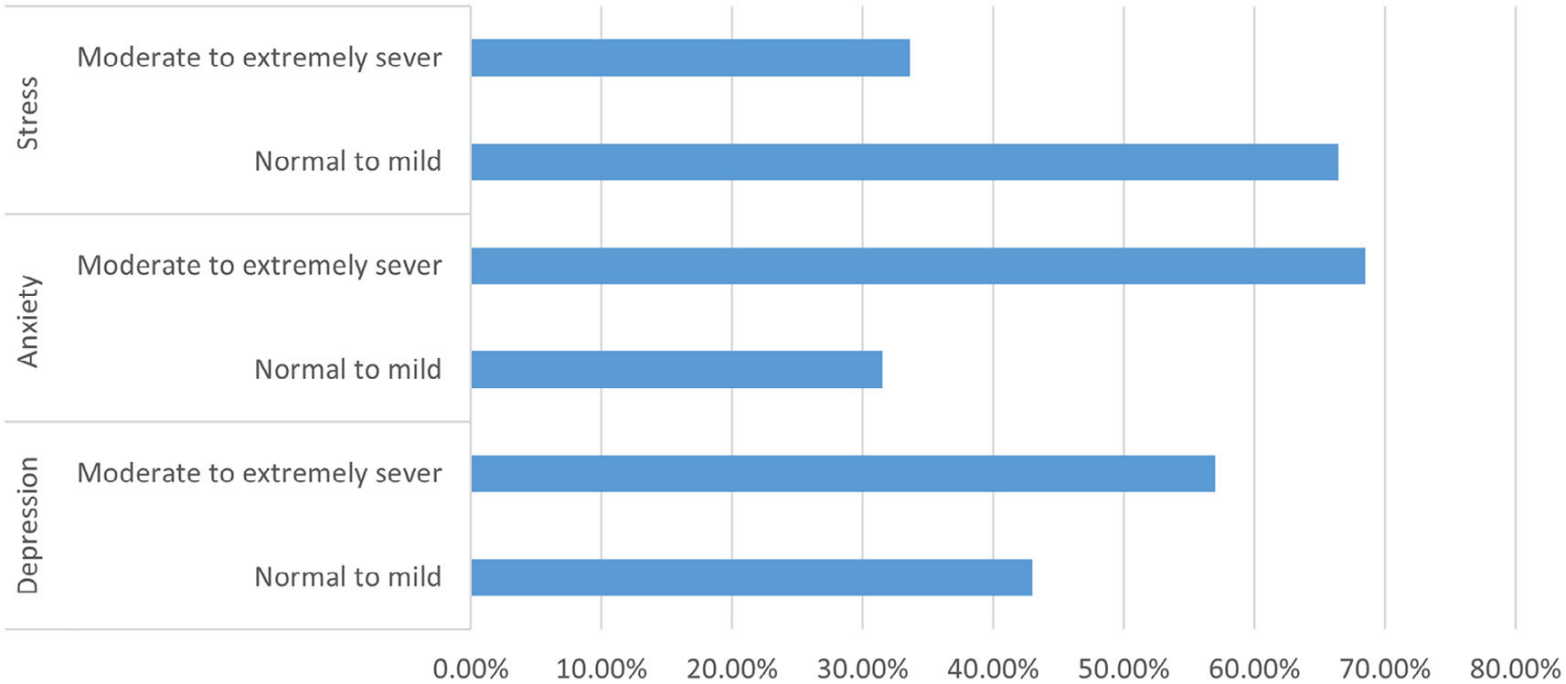
Depression, anxiety, and stress scale (DASS).

Overall, the identified assessment tools indicated that more than half of the participants, 170 (57.0%), had moderate to extremely severe depression, 204 (68.5%) had moderate to extremely severe anxiety, and 100 (33.6%) had moderate to extremely severe stress. Figure 3 provides insight from the study’s adoption of the Depression, anxiety, and stress scale (DASS).

### Association between DASS and demographic characteristics

Table 3 shows the association between DASS scores and demographic characteristics. There were no significant differences in depression and anxiety levels between genders. However, a significant association was found for stress, with 35.6% of females experiencing moderate to highly severe stress compared to 25.8% of males (p < 0.001). Students from the Dental College had the highest rates of moderate to extremely severe anxiety (75.0%) and stress (32.4%) compared to other colleges (p < 0.001 for both). First-year students had the highest prevalence of moderate to extremely severe depression (60.7%), anxiety (69.2%), and stress (26.2%) across all.

**Table 3.** Association between DASS items with demographic characteristics and AI usage among the students.

		Depression				P-value	Anxiety				P-value	Stress				P-value
		Normal to mild		Moderate to severe			Normal to mild		Moderate to severe			Normal to mild		Moderate to extremely severe		
		N	%	N	%		N	%	N	%		N	%	N	%	
Gender	Female	103	43.6%	133	56.4%	0.612	76	32.2%	160	67.8%	0.341	152	64.4%	84	35.6%	**<0.001**
	Male	25	40.3%	37	59.7%		18	29.0%	44	71.0%		46	74.2%	16	25.8%	
Age (Mean ± SD)		21 ± 3		21 ± 2		0.711	21 ± 2		22 ± 3		0.611	21 ± 3		21 ± 2		0.630
Collage	Dental	27	39.7%	41	60.3%	0.221	17	25.0%	51	75.0%	**<0.001**	46	67.6%	22	32.4%	**<0.001**
	Medicine	57	47.5%	63	52.5%		47	39.2%	73	60.8%		81	67.5%	39	32.5%	
	Nursing	33	40.2%	49	59.8%		20	24.4%	62	75.6%		55	67.1%	27	32.9%	
	Pharmacy	11	39.3%	17	60.7%		10	35.7%	18	64.3%		16	57.1%	12	42.9%	
Year of Study	First	42	39.3%	65	60.7%	**<0.001**	33	30.8%	74	69.2%	**<0.001**	79	73.8%	28	26.2%	**<0.001**
	Second	4	21.1%	15	78.9%		0	0.0%	19	100.0%		6	31.6%	13	68.4%	
	Third	43	51.2%	41	48.8%		29	34.5%	55	65.5%		54	64.3%	30	35.7%	
	Fourth	31	42.5%	42	57.5%		28	38.4%	45	61.6%		49	67.1%	24	32.9%	
	Fifth	8	53.3%	7	46.7%		4	26.7%	11	73.3%		10	66.7%	5	33.3%	

The association between depression, anxiety, stress levels, and students' demographic characteristics and AI usage, using Chi-square and Independent samples T-tests. P value is significant if less than 0.05; SD = Standard deviation.

### Association between AI chatbot usage and dASS

Table 4 illustrates the association between AI chatbot usage and DASS scores. Participants who had never spoken with an AI chatbot were more likely to have moderate to extremely severe depression (N = 125, 63.5%) compared to those who had not used an AI chatbot (N = 45, 36.7%, p < 0.001). Additionally, 153 participants (75.0%) who used AI chatbots had moderate to extremely severe anxiety, while only 51 non-users (55.0%) had this level of anxiety (p < 0.001). However, no significant association was found between AI chatbot usage and stress levels (p = 0.236).

**Table 4.** Association between AI usage with demographics and DASS items.

Variables			Have you ever spoken with an artificially intelligent chatbot?				P-value
			Yes		No		
			N	%	N	%	
Demographics	Gender	Male	46	74.2%	16	25.8%	0.231
		Female	160	67.8%	76	32.2%	
	Age (Mean ± SD)		21 ± 3		21 ± 2		0.611
	Collage	Medicine	88	73.3%	32	26.7%	**<0.001**
		Dental	54	79.4%	14	20.6%	
		Pharmacy	14	50.0%	14	50.0%	
		Nursing	50	61.0%	32	39.0%	
	Year of Study	First	60	56.1%	47	43.9%	**<0.001**
		Second	13	68.4%	6	31.6%	
		Third	62	73.8%	22	26.2%	
		Fourth	57	78.1%	16	21.9%	
		Fifth	14	93.3%	1	6.7%	
DASS	Depression	Normal to mild	81	63.3%	47	36.7%	**<0.001**
		Moderate to severe	125	73.5%	45	26.5%	
	Anxiety	Normal to mild	53	56.4%	41	43.6%	**<0.001**
		Moderate to severe	153	75.0%	51	25.0%	
	Stress	Normal to mild	134	67.7%	64	32.3%	0.236
		Moderate to extremely severe	72	72.0%	28	28.0%	

The correlation between AI chatbot engagement, demographic details, and DASS scores among students was examined using Chi-square and Independent Samples T-tests. A P value is significant if it is less than 0.05; SD = Standard deviation.

### Factors predicting AI chatbot usage

The results of the multivariate regression analysis identify factors predicting AI chatbot usage. After adjusting for covariates, students from the College of Medicine were more likely to use AI chatbots than those from the College of Nursing (OR = 3.094, 95% CI: 1.057-3.059, p = 0.039). Additionally, higher levels of depression (OR = 1.022, 95% CI: 1.01-1.085, p < 0.001) and anxiety (OR = 1.05, 95% CI: 1.01-1.21, p < 0.001) were significantly associated with increased AI chatbot usage ( Table 5).

**Table 5.** Multivariate regression analysis of factors predicting AI usage.

	OR	95% CI of the OR		P-value
Gender (Female)	0.541	0.270	1.083	0.083
Age	0.942	0.942	0.829	0.359
	Collage			
Nursing (Reference)	1			
Medicine	3.094	1.057	3.059	**0.039**
Dental	1.080	0.325	3.586	0.900
pharmacy	1.700	0.670	4.314	0.264
	Year of Study			
First (Reference)	1			
Second	5.255	2.634	4.552	<0.001
Third	1.377	0.139	1.024	0.056
Fourth	2.140	0.920	4.981	0.078
Fifth	1.637	0.179	2.268	0.486
Stress	.961	0.904	1.021	0.201
Anxiety	1.05	1.01	1.21	**<0.001**
Depression	1.022	1.01	1.085	**<0.001**

Multivariate regression analysis to identify factors predicting AI usage among students, highlighting odds ratios and confidence intervals; OR = Oddis ratio; CI = Confidence interval.

## Discussion

Alowais et al. (2023) explains that the rapid advancement of AI has ushered in a new era of digital communication tools, including AI-powered chatbots. These chatbots are increasingly employed across various domains, including education and healthcare, to provide information, support, and interaction ( Bajwa et al., 2021). As such, understanding the factors that influence the usage of AI chatbots, particularly among university students, is crucial. This demographic often faces unique academic and social pressures, which may drive their interaction with technological aids ( Tian et al., 2024). Herein, we aimed to investigate the relationship between mental health issues among students in medical and health sciences disciplines and AI chatbot interaction.

This is timely as The World Health Organisation (2022) estimates that the majority of individuals with mental illness do not seek treatment, citing reasons as concerns a perceived damaging of their family’s reputation, proposals for marriage, social status, encountering discrimination, exclusion from communities, and stigma. Consequently, these individuals can experience poor academic achievement ( Bruffaerts et al., 2018) and diminished self-esteem ( Stuart et al., 2019). The findings of this study collaborate this earlier research and underscore a significant prevalence of mental health challenges, including depression, anxiety, and stress, among students in medical and health sciences disciplines, with more than half of the participants reporting moderate to extremely severe symptoms. The findings indicate that AI chatbot usage was associated with higher levels of depression and anxiety. Specifically, students who had interacted with AI chatbots exhibited a greater likelihood of experiencing moderate to extremely severe depression, although no significant correlation was found with stress levels.

The study revealed that the prevalence of mental health issues among university students, particularly in the medical and health sciences fields, is consistent with a substantial body of existing literature outside of the Middle East. Numerous studies by researchers such as Rtbey et al., (2022); Agyapong-Opoku et al., (2023); Ibrahim et al., (2024); Nair et al., (2023) have highlighted the high rates of depression, anxiety, and stress experienced by these healthcare students. in these disciplines, often attributed to the rigorous academic demands and intense competition inherent in healthcare education. The students experiencing these stressful life events, so often a consequence of rigorous academic growth sought support from AI chatbots. This process demonstrates evidence of the presence of Salutogenesis. Originally developed by Antonovsky, salutogenesis explains how some individuals utilize resources available to them to survive and thrive effectively in adverse social conditions ( Antonovsky, 1979; Mottershead et al., 2024). This adoption of AI chatbot platforms appears to demonstrate that health and well-being cannot be conceptualized in the narrowest sense as a biological function. The students appear to be adopting this technology in an attempt to enhance their quality of life and to support them within their adverse circumstances of life within higher education as also highlight by Bani Issa et al. (2024). The authors therefore emphasize that salutogenesis has an important role in creating insight into the mental well-being of students and creating further understanding around AI use within their lives.

This understanding is furthered within the study’s observation that participants perceived medical chatbots as possessing numerous advantages, such as anonymity, convenience, and expedited access to relevant information. The participants appeared to be equally inclined to share emotions and information with a chatbot as they would with a human counterpart. The intriguing aspect is that interactions with chatbots and humans exhibited similar degrees of perceived understanding, intimacy of disclosure, and cognitive reappraisal, indicating that users engage psychologically with chatbots as they do with humans. The study’s participants mentioned using AI chatbots because they felt an understanding with them, that they (participant) can access them (chatbot) at any time which appeared to enhance a sense of familiarity due to the convenience that the chatbot was meeting their immediate needs. This appeared to foster a sense of belonging towards the chatbot and social cohesion mirroring similar relations identified as ‘friendship’ and that this relationship was able to alleviate their feeling of stress which in turn enhancing a bond of trust with the AI chatbot as the participants did not feel that they could or would be judged by the AI chatbot.

Regarding AI chatbot usage and its association with mental health well-being, our findings are consistent with research by Klos et al. (2021), which had suggested a potential link between excessive digital technology use and a consequential adverse negative impact on mental health. Interestingly, the authors highlight this study’s findings that indicate a significant association between AI chatbot usage paralleled with higher levels of depression and anxiety among students. This maybe explained that those students with negative ill-health are seeking support from the AI chatbots rather than the digital exposure is having an adverse effect on their mental health. There appeared to be a lack of a significant relationship with stress levels associated with AI chatbot use which contrasts with findings from studies such as that by Klos et al. (2021). This discrepancy may be attributed to variations in study methodologies, sample characteristics, and the specific platforms or types of AI chatbots examined. It does however, underscore the need for further research to create an understanding about the nuanced interactions between technology use and mental health outcomes in higher educational settings.

The study found no significant association between gender and AI chatbot usage, whilst other studies outside the Middle East have reported gender differences in technology adoption patterns ( Truong et al., 2023). Moreover, the lack of significant association between age and AI chatbot usage in our study contrasts with findings by Truong et al. (2023), which identified age as a moderating factor of medical mobile applications. These discrepancies may stem from variations in sample characteristics, cultural contexts, or the specific types of technology examined, highlighting the need for further investigation into the nuanced factors influencing technology adoption among different global populations.

The high prevalence of AI chatbot engagement aligns with studies indicating increased acceptance and utilization of digital mental health interventions among young adults. Specifically, the popularity of platforms like Snapchat for accessing AI chatbots resonates with research demonstrating the widespread use of social media for mental health-related activities, including seeking support and sharing personal experiences ( Klos et al., 2021). This would suggest that integrating AI chatbots into familiar social media platforms may enhance accessibility and acceptability among students, potentially addressing barriers to traditional mental health services. Whilst this may be favorable the authors note a need for rigor of data and the interpretation of data provided by the AI chatbot. Similarly, to the findings of Ahmed et al., (2025) it is recommended that there is a need to conduct a training program on AI usage in healthcare as well as ensuring that students are aware of the limitations of AI chatbot. This proposed training program could enhance the effectiveness of the usage of AI chatbot platforms whilst ensuring supportive mental health strategies. However, as highlighted by Nawaz et al., (2024) whilst there is indeed evidence of how digital systems can support mental health via enhanced social support, reducing stigma and isolation. Indeed, Mottershead and Ghisoni (2021) demonstrate the opportunities exist for non-pharmaceutical interventions however, a challenge is that the current healthcare landscape appears unprepared for its implementation and clearly there is a need for more explorative studies.

Despite the high prevalence of AI chatbot usage, our study also revealed alarming rates of moderate to extremely severe depression, anxiety, and stress among students highlighting the mental health challenges faced by university populations within the 21 ^st^ century. The continued presence of mental health problems does raise questions about the effectiveness of AI chatbots in mitigating mental health symptoms among students when usage is so high. The authors believe that future research should explore the integration of AI chatbots with other forms of validated and accredited mental health support to optimize outcomes and ensure comprehensive care for this vulnerable population entrusted with our society’s future healthcare needs.

### Implications for practice

University counselling and nursing services can integrate regulated, evidence-informed chatbots as adjuncts within stepped-care (e.g., psychoeducation, symptom monitoring, CBT-style exercises), while ensuring clear guardrails on privacy, escalation, and clinical oversight. Screening using brief tools (e.g., DASS-21) at key academic milestones could trigger low-threshold digital supports and fast-track access to human services when indicated. Embedding chatbot links in familiar student platforms may lower stigma-related barriers. Programs should include student training on appropriate chatbot use and limitations, with routine safety checks (e.g., crisis routing).

### Limitations of the study

Integrating a well-structured demographic and psychological assessment enhances the reliability of our findings. However, there are limitations to consider. The study’s cross-sectional design restricts our ability to establish causality between mental health issues and AI chatbot usage. Undoubtedly, the author’s own lived experience and subjectivity may have influenced the interpretation of these findings, as highlighted by Blaikie (2007). However, precautions were taken to limit the impact of this bias, where possible, by adhering to a clear and robust methodological framework. The sample is limited to a single institution, which may affect the generalizability of the results to broader university populations. However, the data adds a new cultural context from the United Arab Emirates, contributing to global knowledge of this topic. The authors would recommend that future studies could benefit from longitudinal designs and broader demographic sampling to overcome these noted limitations.

## Conclusion

Most of the participants experienced moderate to extremely severe symptoms. Notably, students who had used AI chatbots were more likely to have higher levels of depression and anxiety compared to non-users. Factors such as being a medical student and having a higher academic year were also associated with increased AI chatbot usage. These findings underscore the need for comprehensive mental health interventions and support services tailored to the unique needs of this population, which may include the judicious integration of AI-powered chatbots as part of a broader mental health strategy. In determining the relevance for clinical practice, the use of AI chatbots holds great potential in identifying and treating mental health issues like anxiety, depression, and stress in students and adolescents. Clinical nurses may recommend these technologies as primary support for clients who may not seek in-person support. It is feasible that College based counselling services could utilize AI Chatbots. This could let users monitor their symptoms in real-time and guide them through evidenced based and accredited cognitive behavioral therapy (CBT) treatment. The availability of Chatbots’ twenty-four hours a day and seven days a week, could have a significant positive impact on mental health care within universities and wider societies as AI Chatbots assist with this generations instant demand for a response and rapid assistance. It is feasible that University counsellors as well as wider healthcare professionals could incorporate chatbots into treatment plans, offering enhanced patient and family involvement and therefore, hope and optimism for holistic care and enhanced outcomes.

### Ethical considerations

The study was conducted as per the relevant ethical guidelines and regulations, including the Declaration of Helsinki. After getting approval from RAK College of nursing REC (RAKCON-REC-01-2023/24-F-M) for the study, written informed consent was obtained from the participants. The privacy of the participants and the confidentiality of the collected data were assured.

## Data Availability

In adherence to regulatory practices on the sharing of confidential student data, readers are requested to direct requests for access to the corresponding author – richardm@seha.ae. Data will be shared upon request.
