# Supplementary material for: New Horizons in Higher Education: Examining the Mental Well-Being of Medical & Health Sciences Students Through the Use of Artificial Intelligence Based Chatbot Platforms in the United Arab Emirates – A Cross-Sectional Comparative Study
Source: F1000Res. 2025 Oct 6;14:665. Originally published 2025 Jul 7. [Version 2] doi: 10.12688/f1000research.166372.2 (PMC12504930; doi:10.12688/f1000research.166372.2)
Supplement: Peer review history

# Reviewer report: Reviewer response for version 2

**DOI:** 10.5256/f1000research.188737.r420821

**Published:** 2025-10-07

**Authors:** Assisst .Prof Dr Iman Hussein Alwan

**Affiliations:** 1 psychiatric Mental Health Nursing, University of Baghdad/ College of Nursing, Baghdad, Baghdad Governorate, Iraq

I have reviewed the revised version of the manuscript and confirm that all previous comments and concerns have been adequately addressed by the authors. The revisions have improved the clarity, methodological transparency, and overall presentation of the study. I have no further comments to make, and I consider the article suitable for publication.

Is the work clearly and accurately presented and does it cite the current literature?

Yes

If applicable, is the statistical analysis and its interpretation appropriate?

Yes

Are all the source data underlying the results available to ensure full reproducibility?

Yes

Is the study design appropriate and is the work technically sound?

Yes

Are the conclusions drawn adequately supported by the results?

Yes

Are sufficient details of methods and analysis provided to allow replication by others?

Yes

Reviewer Expertise:

,  community Emotional Intelligence, psychiatric mental health, Psychological , educational, a ddication

I confirm that I have read this submission and believe that I have an appropriate level of expertise to confirm that it is of an acceptable scientific standard.

# Reviewer report: Reviewer response for version 1

**DOI:** 10.5256/f1000research.183349.r399583

**Published:** 2025-08-18

**Authors:** Professor David Taylor

**Affiliations:** 1 Gulf Medical University, Ajman, United Arab Emirates

*For the sake of clarity this report uses the “WADFISH” Scheme.*

*WHY is this interesting?*

 The mental health and well-being of students of the health professions is of crucial importance in maintaining recruitment into the health professions, and of course, for the students themselves. Increasing pressures within the workforce, and increasing student numbers, means that the time available for interpersonal contact between faculty and student is limited.  One option used by the current generations of students is to resort to AI driven chatbots.  This paper examines the extent and potential consequences of that approach.

*What was their AIM?*

 The author’s aim was to identify the relationship between AI Chatbot usage and  Stress, Anxiety and Depression levels among Health Sciences University students. Specifically their research questions were:

 RQ1. What are the frequencies of using the Chatbot among medical and health sciences university students?

 RQ2. What are the reasons for the usage of AI Chatbot to cope with depression, anxiety, and stress among Medical and Health Sciences Students?

 RQ3. Is there a relation between the usage of AI Chatbot and depression, anxiety, and stress among Medical and Health Sciences University Students?

 RQ4. Is there a difference between the group who is using Chatbot and the one who does not about depression, anxiety, and stress levels?

*What did they DO?*

 The authors asked a stratified sample of health professions students to complete three inventories categorising their AI chatbot usability (Tool II), their score on the depression anxiety stress scale (DASS-21: Tool III), all related to their demographics (Tool I).  The authors used standard statistical techniques in their analysis. In my opinion the analysis is sound.

*What did they FIND?*

 The most used application was Snapchat.  Participants who used Chatbots were significantly more likely to suffer from moderate to extremely severe depression than those who did not. The authors recognise that this is not necessarily a causal relationship.

*SO WHAT?*

 AI Chatbots have the potential as a “helpful adjunct” to other interventions in helping students passing through mental health difficulties.

*HOW will this affect me, my institution, my students or patients?*

 In my opinion the biggest issue is in helping students to understand more about the use of AI, whilst ensuring the more conventional support systems remain available.

*General Comments*

 This is an interesting study, which has been well thought through and well executed.  There are several spelling/typographical errors which need attention.  “Chatbot” appears to have been automatically corrected to “Chabot” in several places.  More confusingly in the data analysis and management section there appears the following:

 “A logistic regression analysis was performed to assess the association between the categorized DASS scores and **periodontitis** while adjusting for potential confounding factors”. Is there a typographical error there?

Is the work clearly and accurately presented and does it cite the current literature?

Yes

If applicable, is the statistical analysis and its interpretation appropriate?

Yes

Are all the source data underlying the results available to ensure full reproducibility?

Yes

Is the study design appropriate and is the work technically sound?

Yes

Are the conclusions drawn adequately supported by the results?

Yes

Are sufficient details of methods and analysis provided to allow replication by others?

Yes

Reviewer Expertise:

My area of research is higher education, in particular in qualitative studies of lived experience.I have published several studies relating to AI.

I confirm that I have read this submission and believe that I have an appropriate level of expertise to confirm that it is of an acceptable scientific standard.

# Response

**Published:** 2025-09-05

**Authors:** Dr. Richard Mottershead

**Affiliations:** College of Health Sciences, University of Sharjah, Sharjah, Sharjah, United Arab Emirates

On behalf of the research team, I would like to thank you for the time and energy that you have obviously spent on this detailed and comprehensive review of our article. WE have thrived from your positivity and encouragement and it is obvious that you are only too aware of the importance in addressing and understanding students mental health needs within the MENA region. Our subsequent research will be enhanced by assimilating and acknowledging your suggestions and we are confident that our continuing research will be positively affected through your influence within this review.

 Thank you – Dr. Richard Mottershead.

# Reviewer report: Reviewer response for version 1

**DOI:** 10.5256/f1000research.183349.r399588

**Published:** 2025-08-18

**Authors:** Assisst .Prof Dr Iman Hussein Alwan

**Affiliations:** 1 psychiatric Mental Health Nursing, University of Baghdad/ College of Nursing, Baghdad, Baghdad Governorate, Iraq

The article titled *"New Horizons in Higher Education: Examining the Mental Well-Being of Medical & Health Sciences Students Through the Use of Artificial Intelligence Based Chatbot Platforms in the United Arab Emirates – A Cross-Sectional Comparative Study"*

 Reviewer Comments:

 After reviewing the submitted manuscript, I would like to offer the following scientific explanations with the aim of improving the clarity, consistency, and academic contribution of the study. I reviewed all parts of the study, including the abstract, introduction, methodology, results, discussion, and conclusion.

 Abstract:

- The abstract is informative, but would benefit from explicitly stating the tools used for data collection.
- It is recommended to summarize the key statistical findings more clearly.

Introduction:

1. It is recommended to clarify the scientific gap more clearly by comparing Western literature with the Arab/Gulf environment, to highlight the contribution the study makes.
2. The researcher did not precisely define these challenges. Challenges include: lack of resources, a shortage of mental health professionals, social stigma associated with mental health, and difficulty accessing healthcare.
3. Recommendation: Add examples of other digital technologies being piloted in mental health care, and perhaps the growing role of artificial intelligence in mental health treatment in the Middle East.
4. Some global statistics related to mental health, such as the number of individuals affected by depression and anxiety, support a global view of the mental health problem, but these statistics could be better contextualized in more detail with the reality of the Middle East.

Methods:

- The names of the scales used to measure stress, anxiety, and depression are not mentioned.
- The tools should be clearly identified and explained in the methodology section, including their reliability, validity, and cultural adaptation, especially since the study is being conducted in the UAE, where cultural factors may influence results.

There is insufficient demographic information about the sample : such as (Student age group, Ratio of males to females.)

- type of university is vague. if ethical approval permits clarify whether it is a public or private institution. The sample size (298) was mentioned, but the total population size was not mentioned.
- Tool III: DASS-21: Recommendation: The study could include more details on how the tool was administered to participants. For example, were participants given an explanation of how to answer the questions? Were the questions understandable to everyone, especially in a multicultural setting?

- The design is described as "descriptive comparative," while the researcher aims to "determine the relationship."
- Note: It is better to use the term "correlational" or "cross-sectional correlational" if the goal is to examine only correlational relationships.

Result :  The results are presented clearly, with statistical data such as p-values and OR values. However, the interpretation of these results could be made clearer, particularly regarding the relationship between chatbot use and depression/anxiety.

 The measurement tools were mentioned in the results section, whereas they should be clearly described in the methodology section.

 The researcher report that 63.5% of chatbot users experience depression, versus 36.7% of non-users, but offer no explanation. Discussion of causality versus correlation is essential.

 Include a section on the practical significance of findings (e.g., clinical implications, intervention planning)

 Recommendation: Authors should discuss the causal relationship between chatbot use and depression/anxiety. They should explore whether chatbot use causes mental health problems, or whether preexisting medical conditions (such as depression and anxiety) lead to increased chatbot use.

 Authors should include a section explaining the practical significance of the findings, particularly how the use of intelligent chatbots affects mental health outcomes, and the strength of these effects.

Research question

 Q1: Define what constitutes "frequent use" (e.g., how many times per week/month?).

 Q2: Consider expanding the question to include other motivations (e.g., convenience, privacy, stigma avoidance).

 Q3: Consider moderating variables such as severity of symptoms or type of chatbot interaction (text/audio).

  (Setting and participants: 

 Recommendation: It is important to determine how the sample size was calculated more precisely. For example, how was the required number from each college determined? Was there a proportional distribution among the different disciplines?

 RESULT:

 Data visualization can be enhanced by using a graph or chart to visually illustrate the distribution.

 Some explanation could be added about why these specializations (such as medicine, pharmacy, nursing) were chosen in particular and why a specific number of participants from each college was selected.

 There may be additional details about how these robots are used, such as the amount of time students spend interacting with the robots or the type of assistance they request

 Brief Overview

 This manuscript discovers the relationship between mental health symptoms (depression, anxiety, and stress) and the use of AI-based chatbot platforms among medical and health sciences students in the United Arab Emirates. The theme is relevant and appropriate, lecturing the cumulative confidence on digital mental health tools in higher education. The research offerings   important findings on the prevalence of mental health symptoms and the apparent worth of chatbots among students.

 General Comments

 The paper is usually well-written, with a coherent construction and a clearly defined aim. The use of a cross-sectional design is suitable; however, several methodological and structural aspects need improvement. Some inconsistencies   started in the placement of key information, particularly about the measurement tools. The discussion is rich but could benefit from clearer comparisons with studies from similar cultural backgrounds. Despite these apprehensions, the study contributes valuable insights and is possible for journal after revisions.

 Specific Comments

 �� Title and Abstract

- The title is comprehensive and informative.
- The abstract summarizes the study sufficiently but would advantage from more clear mention of the main answers and statistical significance.

�� Introduction

- The introduction successfully presents the topic and its significance.
- It is recommended to clarify early what constitutes “AI chatbot use” (e.g., frequency, purpose, platform).

�� Methodology

- The methodology section should include a full description of the measurement tools (DASS-21 and other scales) with their psychometric properties and validity, instead of first introducing them in the results.
- The sampling technique and inclusion/exclusion criteria need clearer explanation.
- Ethical agreement is stated but could be more detailed in describing participant staffing.

�� Results

- The results are presented logically and are supported by statistical analysis.
- However, tables need clearer formatting (e.g., add degrees of freedom where relevant, ensure consistent formatting of P-values).
- A brief explanation of what the odds ratio implies in practical terms would improve reader understanding.

�� Discussion

- The discussion interprets the findings appropriately and links them with relevant literature.
- The resilience-focused approach a valuable is a useful addition but    it requires clearer integration into the discussion.
- The researchers   state that chatbot users presented higher depression and anxiety levels, but causality cannot be incidental—this should be more explicitly emphasized.

�� Limitations

- The limitations are acknowledged properly.
- The influence of the author’s own interpretation is noted, which validates transparency. However, upcoming studies should consider triangulation or multi-site data to strengthen credibility.

�� Conclusion and Implications

- The conclusion is aligned with the findings and suggests practical applications in clinical and university counseling settings.
- Recommendations for integrating chatbot use into mental health care are promising, but care should be taken not to overstate the current evidence base.

�� Language and Style

- The manuscript is mostly written in clear academic English.
- Some grammatical inconsistencies and redundancy exist and should be addressed in editing (e.g., sentence structure, paragraph transitions).
- Approval Status
   Approved with reservations
  The article addresses an important and timely topic and offers relevant insights into AI chatbot use and student mental health. However, revisions are required in the methodology section (particularly in describing the measurement tools), results formatting, and some language editing. After these improvements, the manuscript will be suitable for acceptance.

Is the work clearly and accurately presented and does it cite the current literature?

Yes

If applicable, is the statistical analysis and its interpretation appropriate?

Yes

Are all the source data underlying the results available to ensure full reproducibility?

Yes

Is the study design appropriate and is the work technically sound?

Yes

Are the conclusions drawn adequately supported by the results?

Yes

Are sufficient details of methods and analysis provided to allow replication by others?

Yes

Reviewer Expertise:

,  community Emotional Intelligence, psychiatric mental health, Psychological , educational, a ddication

I confirm that I have read this submission and believe that I have an appropriate level of expertise to confirm that it is of an acceptable scientific standard, however I have significant reservations, as outlined above.

# Response

**Published:** 2025-09-05

**Authors:** Dr. Richard Mottershead

**Affiliations:** College of Health Sciences, University of Sharjah, Sharjah, Sharjah, United Arab Emirates

On behalf of the research team, I would like to thank you for the time and energy that you have obviously spent on this detailed and comprehensive review of our article. Indeed, we feel confident that our subsequent article is greatly enhanced by assimilating your valid points and guiding our continuing research. 

 Again, thank you and we hope our responses acknowledge the value we place in your peer-review report. Dr. Richard Mottershead 

 Reviewer Comments:

 After reviewing the submitted manuscript, I would like to offer the following scientific explanations with the aim of improving the clarity, consistency, and academic contribution of the study. I reviewed all parts of the study, including the abstract, introduction, methodology, results, discussion, and conclusion.

 Abstract:

 The abstract is informative, but would benefit from explicitly stating the tools used for data collection. Thank you and we were constrained within the word limit within the abstract to omit certain information in order to meet the journal requirements.

 It is recommended to summarize the key statistical findings more clearly. Thank you for your suggestion but the team thought to try to engage with the widest group of readers to elaborate if possible.

 Introduction:

 It is recommended to clarify the scientific gap more clearly by comparing Western literature with the Arab/Gulf environment, to highlight the contribution the study makes. This is a key-interest within the research team and so your point is valid. The challenge to achieve this is that there is often a negative void of literature within one region that always comparisons to be made.

 The researcher did not precisely define these challenges. Challenges include: lack of resources, a shortage of mental health professionals, social stigma associated with mental health, and difficulty accessing healthcare. We will be seeking to expand on these challenges and to explore further within the next publication.

 Recommendation: Add examples of other digital technologies being piloted in mental health care, and perhaps the growing role of artificial intelligence in mental health treatment in the Middle East. We have listed some current examples that are linked to members of the research team but wanted to ensure that we maintained a focus on this study’s aims and objectives.

 Some global statistics related to mental health, such as the number of individuals affected by depression and anxiety, support a global view of the mental health problem, but these statistics could be better contextualized in more detail with the reality of the Middle East. We acknowledge your valid point and are again restrained by the word limit within this particular article.

 Methods:

 The names of the scales used to measure stress, anxiety, and depression are not mentioned.

 The tools should be clearly identified and explained in the methodology section, including their reliability, validity, and cultural adaptation, especially since the study is being conducted in the UAE, where cultural factors may influence results.

 There is insufficient demographic information about the sample : such as (Student age group, Ratio of males to females.) type of university is vague. if ethical approval permits clarify whether it is a public or private institution. The sample size (298) was mentioned, but the total population size was not mentioned. Thank you for your valid points but in order to maintain confidentiality were not able to be too specific. The number of institutions delivering these courses within the UAE is relatively small.

 Tool III: DASS-21: Recommendation: The study could include more details on how the tool was administered to participants. For example, were participants given an explanation of how to answer the questions? Were the questions understandable to everyone, especially in a multicultural setting? There is some discussion of language and an effective method of engaging with the partcipants is established, implied and acknowledged.

 The design is described as "descriptive comparative," while the researcher aims to "determine the relationship."

 Note: It is better to use the term "correlational" or "cross-sectional correlational" if the goal is to examine only correlational relationships. We took direction from previous comparative studies and as we make reference to them we wished to maintain an easy comparison.  

 Result:  The results are presented clearly, with statistical data such as p-values and OR values. However, the interpretation of these results could be made clearer, particularly regarding the relationship between chatbot use and depression/anxiety. Again, there was a need to provide a discussion on all findings and we were conscious not to focus on one point that may limit our ability to discuss all findings.

 The measurement tools were mentioned in the results section, whereas they should be clearly described in the methodology section. We were encouraged to focus this material within the results section as it became a relevant part of the findings of the study.

 The researcher report that 63.5% of chatbot users experience depression, versus 36.7% of non-users, but offer no explanation. Discussion of causality versus correlation is essential.

 Include a section on the practical significance of findings (e.g., clinical implications, intervention planning)

 Recommendation: Authors should discuss the causal relationship between chatbot use and depression/anxiety. They should explore whether chatbot use causes mental health problems, or whether preexisting medical conditions (such as depression and anxiety) lead to increased chatbot use.

 This is a valid point and we thank you for raising this point. The emergence of causality was discussed by the participants and attributed to factors that they themselves  identified. As the study was concerned to allow the participants to share their voice/narrative we relied on the participants mean making process of their experiences.

 Authors should include a section explaining the practical significance of the findings, particularly how the use of intelligent chatbots affects mental health outcomes, and the strength of these effects.

The limitations of article length curtails such an expansive desire to fully explore all points.

 Research question

 Q1: Define what constitutes "frequent use" (e.g., how many times per week/month?).

 Q2: Consider expanding the question to include other motivations (e.g., convenience, privacy, stigma avoidance).

 Q3: Consider moderating variables such as severity of symptoms or type of chatbot interaction (text/audio).

  (Setting and participants:

 Recommendation: It is important to determine how the sample size was calculated more precisely. For example, how was the required number from each college determined? Was there a proportional distribution among the different disciplines?

 Thank you for your suggestions and as in so many cases data saturation is the key determining factor.

 RESULT:

 Data visualization can be enhanced by using a graph or chart to visually illustrate the distribution.

Thank you, we will look at this suggestion within the next paper.

 Some explanation could be added about why these specializations (such as medicine, pharmacy, nursing) were chosen in particular and why a specific number of participants from each college was selected.

Thank you and we believe an explanation of these being the colleges within the specified institution provides an explanation.
